# Estimating the Timing of Mother-to-Child Transmission of the Human Immunodeficiency Virus Type 1 Using a Viral Molecular Evolution Model

**DOI:** 10.1371/journal.pone.0090421

**Published:** 2014-04-09

**Authors:** Antoine Chaillon, Tanawan Samleerat, Faustine Zoveda, Sébastien Ballesteros, Alain Moreau, Nicole Ngo-Giang-Huong, Gonzague Jourdain, Sara Gianella, Marc Lallemant, Frantz Depaulis, Francis Barin

**Affiliations:** 1 Université François-Rabelais, Institut National de la Santé et de la Recherche Médicale - Unité 966 et Laboratoire de Virologie, Centre Hopsitalier Universitaire Bretonneau, Tours, France; 2 University of California San Diego, Department of Pathology, San Diego, California, United States of America; 3 Faculty of Associated Medical Sciences, Chiang Mai University, Chiang Mai, Thailand; 4 Laboratoire Ecologie et Evolution, Centre National de la Recherche Scientifique - Unité Mixte de Recherche 7625- Ecole Normale Supérieure, Paris, France; 5 Institut de Recherche pour le Développement, Chiang Mai, Thailand; 6 Harvard School of Public Health, Department of Immunology and Infectious Diseases, Boston, Massachusetts, United States of America; Institut Pasteur, France

## Abstract

**Background:**

Mother-to-child transmission (MTCT) is responsible for most pediatric HIV-1 infections worldwide. It can occur during pregnancy, labor, or breastfeeding. Numerous studies have used coalescent and molecular clock methods to understand the epidemic history of HIV-1, but the timing of vertical transmission has not been studied using these methods. Taking advantage of the constant accumulation of HIV genetic variation over time and using longitudinally sampled viral sequences, we used a coalescent approach to investigate the timing of MTCT.

**Materials and Methods:**

Six-hundred and twenty-two clonal *env* sequences from the RNA and DNA viral population were longitudinally sampled from nine HIV-1 infected mother-and-child pairs [range: 277–1034 days]. For each transmission pair, timing of MTCT was determined using a coalescent-based model within a Bayesian statistical framework. Results were compared with available estimates of MTCT timing obtained with the classic biomedical approach based on serial HIV DNA detection by PCR assays.

**Results:**

Four children were infected during pregnancy, whereas the remaining five children were infected at time of delivery. For eight out of nine pairs, results were consistent with the transmission periods assessed by standard PCR-based assay. The discordance in the remaining case was likely confused by co-infection, with simultaneous introduction of multiple maternal viral variants at the time of delivery.

**Conclusions:**

The study provided the opportunity to validate the Bayesian coalescent approach that determines the timing of MTCT of HIV-1. It illustrates the power of population genetics approaches to reliably estimate the timing of transmission events and deepens our knowledge about the dynamics of viral evolution in HIV-infected children, accounting for the complexity of multiple transmission events.

## Introduction

The prevention of mother-to-child transmission (MTCT) of the human immunodeficiency virus (HIV) is one of the main global health issues and is one of the World Health Organization's key priorities. HIV-1 still infects about two million children worldwide, with 330,000 new infections reported in children in 2011 (Report on the Global AIDS Epidemic 2012, Geneva, World Health Organization). Without preventive intervention, probability of MTCT is 15–30% in high-income countries and up to 25–40% in sub-Saharan Africa, mainly due to differences in infant feeding practices [1]. In non-breastfeeding populations, and in the absence of chemoprophylaxis and targeted obstetrical prevention measures, transmission occurs *in utero* in approximately one third of the cases (mostly late in the third trimester of pregnancy) and *intrapartum* in the remaining two thirds [1], [2].

Application of various phylogenetic approaches to HIV-1 has led to reconstruction of HIV-1 evolutionary history within and between hosts with good precision [3]. At the epidemiological level, history and origin of the HIV-1 epidemic have been successfully explored through molecular phylogeny [4]–[6], and these approaches also provided insights into clusters of HIV-1 transmission [7]–[9]. Moreover, application of these molecular evolutionary processes at the intra-host scale were implemented to investigate HIV-1 compartmentalization dynamics, disease progression, and adaptive response to drug therapy [10]–[14]. If traditional phylogenetic analyses establish evolutionary relatedness, recent developments that incorporate sample date information have permitted new approaches [15]. Specifically, the introduction of molecular clock in phylogenetic inference allowed more precise investigations into the evolutionary processes in order to infer molecular phylodynamics of HIV-1 [16]. Additionally, longitudinally obtained sequences from patients have been more recently used to investigate transmission direction and to estimate date of infection [17]–[21].

Aside from the descriptions of rare early *in utero* transmission events [22], the timing of transmission has been mostly estimated using partially indirect dynamic models [2]. Such probabilistic approaches exclusively rely on the virus detection at successive sampling time points after delivery [23]. Molecular variability constitutes valuable additional information, and viral sequences sampled at different time points can be used to investigate the rate and population dynamics of intra-host HIV-1 evolution [6], [24]–[30]. We hypothesized that this approach could provide a good approximation of the timing of MTCT by estimating the time of most recent common ancestor (TMRCA) of viruses in the child. In this study, we used a coalescent approach within a Bayesian statistical framework to investigate the timing of MTCT. Using time series of cellular HIV-1 DNA and plasma HIV-1 RNA *env* sequences from nine mother-child pairs, we compared these quantitative results with the classic biomedical definition based on serial PCR that more qualitatively discriminates between transmission periods (i.e. *in utero vs. intrapartum*).

## Materials and Methods

### Ethics Statement

The initial protocol and its amendments were approved by the ethics committees of the Thai Ministry of Public Health, Chiang Mai University, and the Harvard School of Public Health. All study sites complied with regulations of the Department of Health and Human Services for the protection of research subjects. Women were enrolled at 28 weeks' gestation if they provided written informed consent.

### Biological material

Nine mother-infant pairs, enrolled in the Perinatal HIV Prevention Trial-1 (PHPT-1) in Thailand were studied [31].The infants were not breastfed. HIV-1 infection status was determined by PCR-based HIV-1 proviral DNA detection, using DNA extracted from peripheral blood collected longitudinally within 48 hours of birth, at six weeks, four months, and six months of age. We considered the infant to have been infected *in utero* if the first test (using blood collected within 48 hours of birth) was positive and *intrapartum* if the first test result was negative but the following test results were positive [23]. HIV-1 *env* gene sequences were obtained from the mothers at delivery and from sequential samples collected at different times in their babies starting from the first positive PCR result up to several months or several years after birth (table 1). Extraction, amplification, cloning, and sequencing of *env* genes were done as previously described [32]. Briefly, genomic DNA was extracted from peripheral blood and viral RNA was extracted from plasma samples. A 1.2 kb fragment covering almost the entire HIV-1 *env* gp120 gene (from upstream V1 to downstream V5) was amplified by nested polymerase chain reaction (PCR) or PCR following reverse transcription (RT-PCR) using subtype-specific primers [32]. For each sample, several independent PCRs were pooled before cloning and sequencing in order to be representative of the viral diversity. All sequences were deposited in GenBank under accession numbers HM121341 to HM121962. A preliminary phylogenetic analysis using reference sequences from all the major HIV-1 clades showed that all individuals were infected by CRF01_AE strains, the predominant lineage in Thailand.

**Table 1:** Pairs sampling informations.

Pair ID (IU/IP*)	Mother (M)/Infant (I)	Time points (days after delivery)/DNA (D) or RNA (R)/number of clones								Total sequences
		1	2	3	4	5	6	7	8	
**0779 (IP)**	M	0/D/15								84
	I		124/R/7	224/D/10	278/D/10278/R/10	376/R/9	852/D/4	936/R/10	1034/R/9	
**0858 (IU)**	M	0/D/12								92
	I	0/R/14	128/D/10128/R/4	183/D/10	370/D/10	482/D/7482/R/9	575/D/10	860/D/6		
**0939 (IP)**	M	0/D/6								55
	I		44/R/6	126/D/10	187/D/11	231/D/11	861/D/11			
**1005 (IU)**	M	0/D/230/R/9								101
	I	0/D/10	47/R/17	122/D/10	185/D/10	355/R/11	543/D/11			
**1021 (IP)**	M	0/D/6								56
	I		68/R/6	124/R/10	429/R/9	551/D/5	611/R/10	820/R/10		
**1110 (IU)**	M	0/D/8								29
	I	0/R/10	475/D/11							
**1224 (IU)**	M	0/D/15								65
	I	0/R/11	122/D/11122/R/8	185/D/10	277/D/10					
**1333 (IP)**	M	0/D/11								66
	I		87/D/1187/R/11	115/D/9	185/D/8185/R/10	350/D/6				
**1391 (IP)**	M	0/D/11								74
	I		47/R/10	128/D/9128/R/10	184/D/9	459/R/10	526/D/8	755/D/7		

IU: *in utero* transmission - IP: *intrapartum* transmission.

### Data analyses

**Figure 1:**
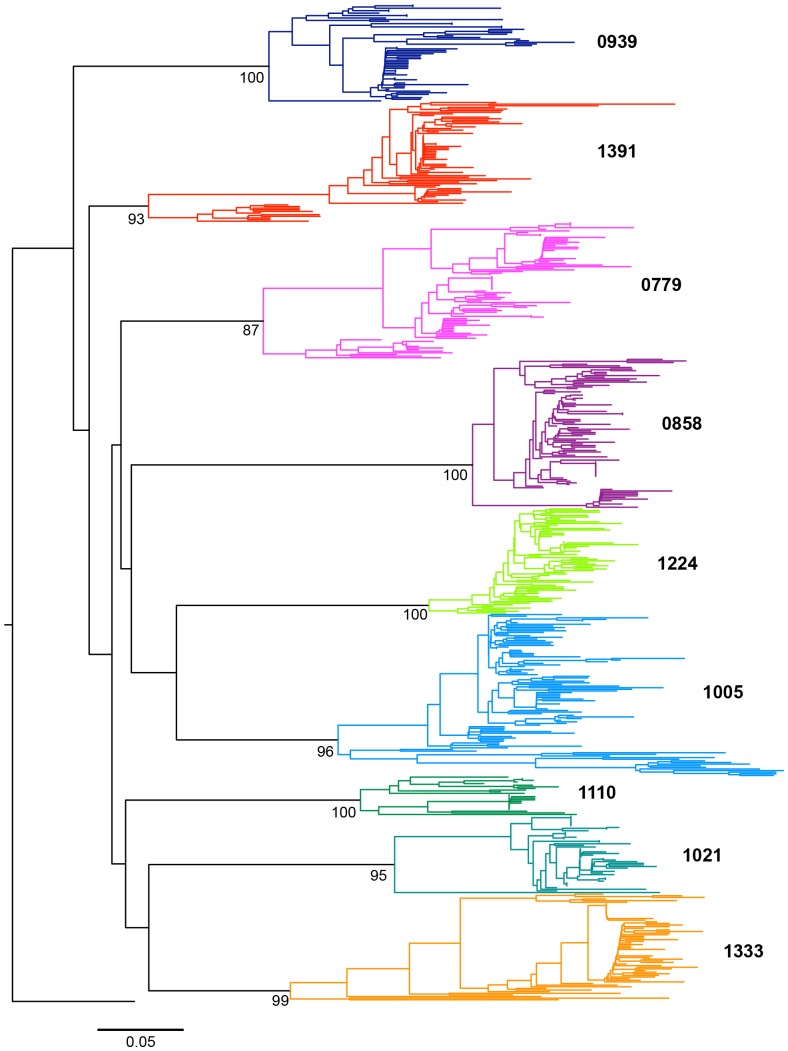
Rooted phylogenetic tree of the entire dataset (mothers and children of the nine pairs), reconstructed specifying a GTR+Gamma model. Each color represents a different pair. The external group (black) belongs to the CRF01_AE subtype. The scale corresponds to 5% of divergence between sequences. The bootstrap values are indicated.

Sequences were aligned with the MAFFT v7.13 software [33] in both multiple and pairwise alignments, using an HIV-B and a consensus of HIV-1 CRF01_AE sequence as reference sequences. The resulting alignments were visually inspected with JALVIEW v2.0 [34]. The partition of genetic variation (diversity) was investigated through a molecular analysis of variance (AMOVA; [35]) implemented in ARLEQUIN v3.0 [36] to assess which fraction of genetic variation was accounted by various factors: mother-and-child pairs, sampling times, and DNA vs. RNA, (table 2). For the whole dataset of nine mother-and-child pair, a maximum likelihood (ML) tree was constructed using PHYML v2.4 [37] with a GTR+Gamma mutational model as assessed by the mutational model selection procedure implemented in HYPHY v2.2 [38], [39]. Branch support was evaluated with 1000 bootstrap replicates (Figure 1).

**Table 2:** Effect of different factors (mother-infant pair, duration of follow up and DNA/RNA origin of the viral sequences) on molecular variance analyzed by AMOVA.

Data	Molecular variance due to M/I pair relative to duration of follow-up			Molecular variance due to M/I pair relative to origin of sequences (times pooled)	
	DNA	RNA	DNA & RNA		
**Pairs**	81.25%*	77.24%*	79.23%*	**Pairs**	79.02%*
**Time within each pair**	6.47%*	14.93%*	10.06%*	**DNA/RNA within each pair**	3.87%*
**Residual**	12.28%*	7.83%*	10.71%*	**Residual**	17.12%*

*p<0.001.

AMOVA: analysis of the molecular variance.

Finally, to assess the impact of putative recombination on our phylogenetic analyses, we applied the method of Genetic Algorithm implemented in GARD [40] looking for potential topological tree incongruence (i.e. the impact of recombination on tree reconstruction) and tested for significance using Kishino Hasegawa (KH) topological incongruence analysis [41].

### Coalescent Bayesian Markov Chain Monte Carlo (MCMC) estimates of the transmission timing

We used the BEAST package v1.7 [42] to estimate MTCT timing with a combination of probability models of gene trees and Bayesian statistical framework. Analysis were conducted under the best-fitting set of molecular, coalescent and demographic models (i.e. GTR+Gamma and an extended Bayesian skyline plot), as indicated by a Bayes Factor >20 (Table S1) [43], [44]. For each mother infant pair, three chains were run assuming an uncorrelated lognormal relaxed molecular clock that allows the rate of evolution to vary across the tree [15]. Three independent chains were run for each pair with similar prior but different initial states, checking that such replicates reached similar results in order to assess convergence. Mixing and convergence (i.e. effective sample size greater than 200 for all parameters) were assessed in Tracer v1.5. All chains reached stationarity before 50,000,000 steps (except for pair 0779, which analysis had to be rerun with 100,000,000 steps for convergence). The Logcombiner v1.7.5 tool was then used to combine the chains. The first 10% of the chains were discarded (*burn in* period). Subsequently, the chain was regularly sampled to get an average estimate of parameter values and associated uncertainty [95% highest posterior density (HPD)]. Maximum clade credibility tree was selected with the software TREEANNOTATOR v1.7.4, after a 10% burn in. Trees were visualized in FIGTREE v.1.3.1.

## Results

### MTCT timing according to reference diagnostic procedures

The timing of MTCT was estimated using qualitative PCR assays for HIV-1 DNA detection in sequential samples collected from the infants. According to these data, four infants were infected during pregnancy (pairs 0858, 1005, 1110, 1224; *in utero* transmission), whereas five infants were infected at delivery or perinataly (pairs 0779, 0939, 1021, 1333, 1391; *intrapartum* transmission) (table 1).

### Sequences analysis

A mean of 6 sequential samples per infant was included in the study (*range* = 2–8) with a mean follow-up period of 664 days (range: 277–1034 days; table 1). A total of 622 *env* sequences were analyzed, with a mean number of 12 clones per time-point (*n* = 4–32) and a mean of 69 clones for each mother-infant pair (range: 29–101). Among them, 61.4% were issued from cellular DNA, and 38.6% were issued from plasma viral RNA (382 and 240 sequences, respectively; Table 1).

Population structure was explored through an analysis of the molecular variance (AMOVA). The genetic variability between viral sequences (diversity) was explained by order of importance by: (*i*) mother-child pair, (*ii*) mother *vs* infant within each pair, (*iii*) the temporal stratification of the data, and (*iv*) the DNA or RNA origin of the sequences (Table 2). For instance, the percentage of molecular variance attributed to the nature of the sequences' origin (i.e. either cellular DNA or plasma viral RNA) was 3.9%, compared to 10.1% and 79.0% for the temporal stratification of the data and the nature of the pair, respectively. This finding suggests that the origin of the sequences (i.e. cellular DNA or plasma viral RNA) had moderate influence on the results.

Since recombination could strongly influence the population structure of sequences dataset, sequences were screened for recombination before phylogenetic analysis using GARD [45]. Though we found evidenced of single recombination events for most of the pairs, topological incongruence analysis using the KH test was not significant, indicating the signal for recombination was likely due to substitution rate variation, rather than differing phylogenetic history [41].

### Phylogenetic analysis

**Figure 2:**
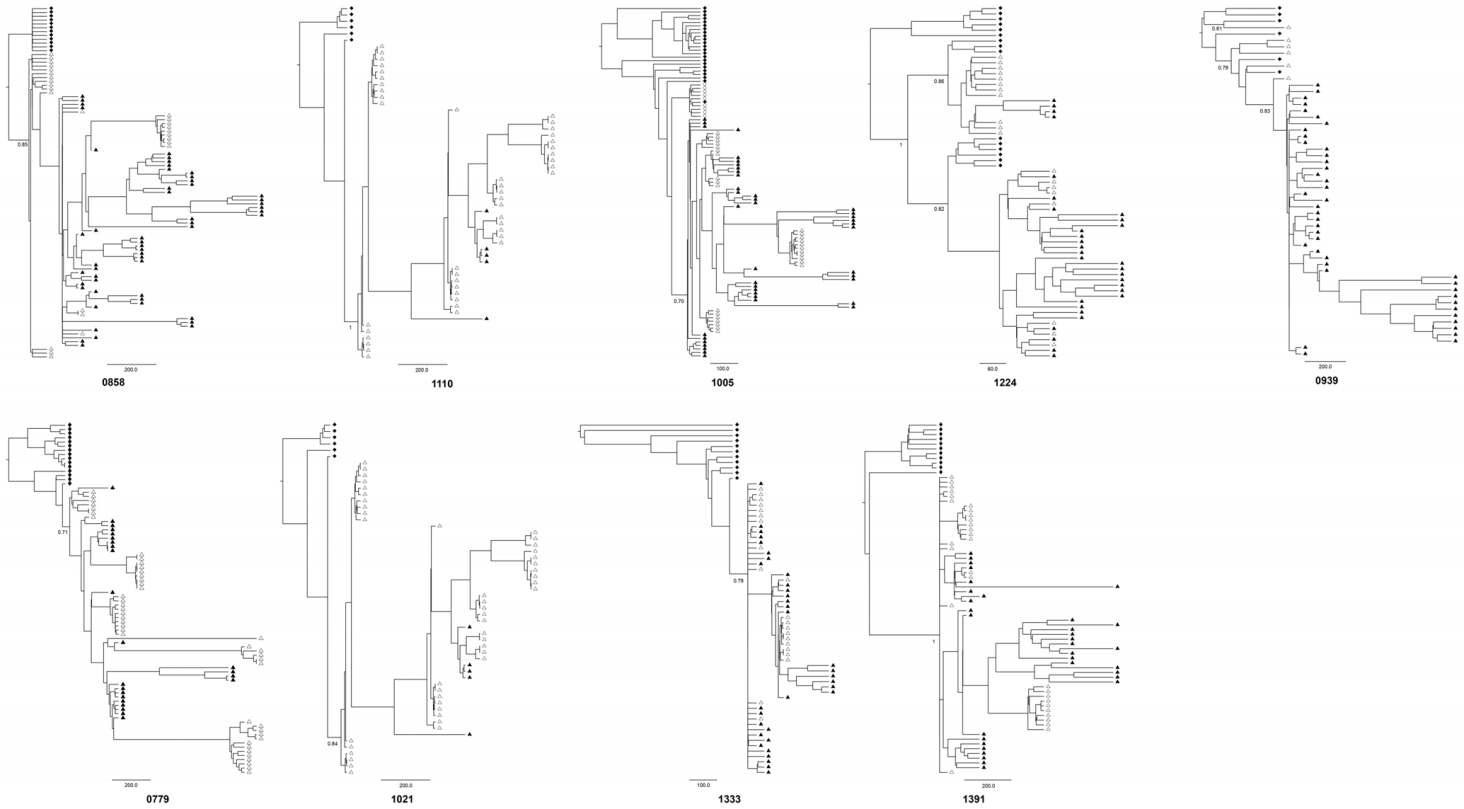
Bayesian MCMC phylogenetic trees. Maternal and child sequences are represented by diamonds and triangles respectively. Filled and unfilled symbols refer to DNA and RNA samples respectively. Time scale expressed in days is indicated below each tree. Posterior probabilities of the main lineages are indicated at the root.

As expected, the phylogeny of the entire data set (Figure 1) showed well supported (monophyletic) subtrees for each mother-infant pair, since each child viral sequence was directly and uniquely derived from the corresponding mother. Each mother-infant tree can therefore be considered separately (Figure 2). Two topological patterns corresponding to two different types of transmission events could be distinguished: eight of nine pairs supported a single viral transmission event with child sequences constituting a well-differentiated (monophyletic) subtree (Figure 2: pairs 0779, 1021, 1333, 1391 0858, 1005 and 1110) and were supported by high posterior probabilities (Figure 2). In these cases, a unique viral ancestor was likely transmitted from the mother to child. In contrast, viral lineages in the infants were intermixed with the mother lineages for pairs 0939, and 1224 (Figure 2). Such polyphyly of the child subtrees can be a consequence of (*i*) a poor phylogenetic resolution or (ii) transmission of several maternal variants. According to this second hypothesis, the TMRCA estimate would trace back to some point during the course of the maternal viral evolution. This kind of pattern would then provide support for multiple transmission variants that may have occurred during a single event (co-infection), or as several successive events (super-infections). Biological data obtained from the reference diagnostic PCR-based procedure may help to distinguish between these possibilities. Infection of infant 0939, which occurred perinatally, might be related to a single transmission event. Indeed, transmission of multiple variants during this time-limited period is more likely to have occurred during a single event (co-infection) rather than successive events (superinfections).

**Figure 3:**
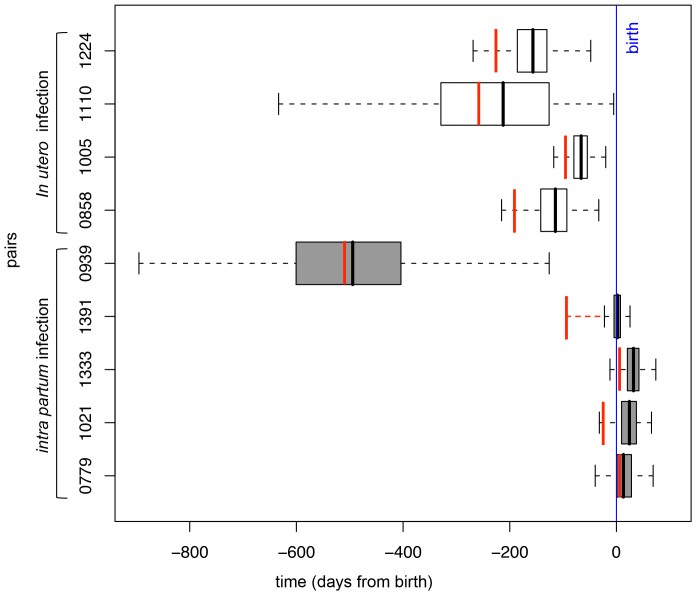
Boxplots of the TMRCA posterior distributions. Dashed lines represent the 95% High Posterior Density (HPD). First (25%) and third (75%) quartiles (limits of the rectangle) and median (thick line) are also indicated. According to the biomedical reference diagnostic method, the four top infants with white boxplots were infected *in utero*, whereas the five bottom infants with grey boxplots were infected *intra partum*. Vertical red lines indicate the maximum estimates of TMRCA.

In contrast, infection of infant 1224, which occurred *in utero*, might have resulted from either a co-infection by several variants during a simple event or super-infections due to several sequential events (Figure 3).

### Coalescent Bayesian Markov Chain Monte Carlo (MCMC) estimate of transmission timing

For the nine pairs, the posterior distribution of the child viral population TMRCA was inferred. The resulting estimate of the TMRCA closely corresponded to the transmission timing according to standard assays (Figure 3). The genealogical inferences were consistent with the biological diagnosis based on timing of HIV-1 proviral detection for eight of nine pairs. The 95% HPD of possible infant root age overlapped birth when viral transmission occurred *intrapartum* according to the reference biological diagnosis (Figure 3: four bottom grey boxes) and excluded it when transmission occurred *in utero* (Figure 3: four top open boxes). Variance of TMRCA largely varied among the pairs. Though the inferred TMRCA results were consistent for five pairs (0779, 1021, 1333, 1391 and 1005; HPD range extending to roughly two months), others showed a greater variance. This variance extended to almost five months for pairs 0858 and 1224. Pair 1110 showed an extremely large range extending close to birth but was documented with only 29 sequences, including 62% of sequences issued from cellular DNA. Of note, pair 0939 range excluded birth and was discordant with the biomedical diagnostic assay. However, the corresponding phylogenetic tree suggested infection with multiple viral variants (Figure 2). The 95% HPD largely preceded birth, extending even before pregnancy, thereby reinforcing the probable infection by several maternal variants. The MRCA of the viral variants should then trace back to the mother's viral population and may even precede pregnancy. If the timing assumed by the biomedical diagnosis was correct, it is possible that several maternal variants were transmitted at birth in child 0939.

**Figure 4:**
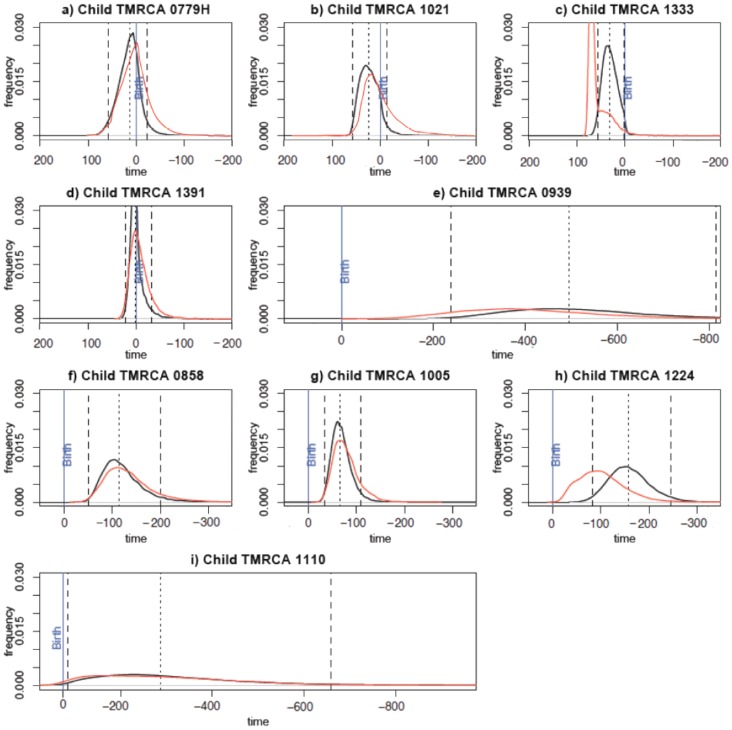
Comparative TMRCA posterior distributions. Red curves correspond to posterior distribution of children-only data. Black curves refer to posterior distribution of pair (mother and child) data. Dashed lines represent the 95% confidence interval. Median (hashed line) is indicated. The blue vertical line indicates birth and times on the x axis are expressed in days since birth. Y axis: TMRCA density; X axis: time in days. a to e: infants infected *intrapartum*. f to i: infants infected *in utero*.

Considering the “children-only” dataset, parameter estimates were still consistent with those obtained with the entire dataset. Inferred TMRCA were mutually highly consistent for 7 of the 9 pairs and in a lesser extent for pairs 1224, 1333 who were also distinct by the shortest duration of follow-up (respectively 277 and 350 days) (Figure 4). This finding was expected considering that the child TMRCA could be estimated regardless of the maternal sequence data (Figure 4).

## Discussion

HIV-1 pediatric infection is a challenging health issue worldwide, and a primary goal of HIV-1 prevention programs is to limit acquisition of HIV-1 during mother-to-child transmission [1]. Although it has been shown that MTCT of HIV-1 can occur both during pregnancy and at delivery [2], the exact timing of MTCT remains difficult to determine precisely with biomedical methods based on serial PCR and indirect probabilistic approaches [23]. To our knowledge, time-measured Bayesian MCMC analyses of longitudinally sampled sequences have never been used to investigate this question. The well-documented sample of longitudinally collected sequences from nine HIV-1 infected mother-infant pairs included in this study represents a unique opportunity to investigate MTCT timing and to validate Bayesian inference.

The maternal and infant viral populations within each pair were significantly differentiated, thus reflecting a drastic bottleneck associated with transmission [32], [46]–[56]. This bottleneck and the subsequent molecular evolution within the infected child provided the basis for our proposed approach. Tree topologies suggested different types of transmission: six pairs showed well-supported monophyletic subtrees claiming for a single transmission event of a single viral variant. In contrast, the data suggested transmission of several variants, as indicated by several separate branches, for the three remaining pairs.

Transmission time estimates using a time-scaled Bayesian phylogenetic approach were shown to be reliable. Despite the short time scales involved (from 277 to 1034 days depending of the mother-infant pair), they were qualitatively highly consistent with the reference diagnostic procedures based on timing of HIV-1 proviral DNA detection. In eight of nine cases, our inferences of the timing of transmission corroborated the PCR results, including four *in utero* and four *intrapartum* infected infants. These results validate the time-scaled Bayesian approach to date MTCT of HIV-1. The single discordant case concerned a pair for which TMRCA was not consistent with the biomedical diagnosis that argued for *intrapartum* transmission. Transmission of multiple maternal variants was strongly suggested by the phylogenetic analysis of this mother-infant pair. The introduction of several variants in a single transmission event or closely related events could lead to large uncertainty in the estimated timing of transmission, leading the ancestry among viral lineages to trace further back in time in the mother's population. However, it remains difficult to distinguish between this hypothesis supported by the PCR results and a possible misclassification by the standard diagnostic assay.

The observed TMRCA variance largely varied among the pairs and could be partially linked to the time of transmission; the more recent the transmission, the more precise the estimate. Hence, TMRCA estimates were consistent (corresponding to a greater coefficient of variance) for five pairs, particularly for the *intrapartum* infected children. Interestingly, a wide confidence interval was observed for two infants (pair 1110, and pair 1224 albeit at a lesser extent), considered to have been infected *in utero* according to the PCR results. This uncertainty might be explained by both the transmission of several variants for the two cases and a low number of available sequences for pair 1110.

We must consider that the transmission of several variants might be attributed either to introduction of several maternal variants in a single transmission event (co-infection) or to several successive introductions of maternal variants (super-infection) that could occur during pregnancy (*in utero*) or during pregnancy and then at delivery (*in utero* and *intrapartum*). Discrimination between these two possibilities remains a challenging objective. Unlike phylogenetic methods that follow a lineage backwards to coalescence, probabilistic models were implemented to detect if transmission occurred because of one or several variants [57]. To our knowledge, superinfection during pregnancy has never been modeled through time. A model introducing migration and divergence processes between the viral populations of the mother and its child might distinguish co-infection from superinfection.

This study was limited by the inclusion of sequences issued from both cellular DNA and plasma viral RNA, which may reflect different tempos of HIV-1 evolution. However, our AMOVA analysis suggested that the origin of the sequences (i.e. cellular DNA or plasma viral RNA) did not play a major role on the observed molecular variance. Additionally, the use of a relaxed clock model partially accommodates for the potential different evolution rate of sequences present in cellular DNA and plasma viral RNA. The reappearance of archived viral population must be considered [58], [59]. For example, the phylogenetic tree of pair 1391 showed that proviral DNA sequences sampled at time *t* = 755 days in the child were extremely close to RNA sequences present earlier in his plasma, at 47 and 184 days after birth. This finding suggests the possibility of continuous re-emergence of variants from putative viral reservoirs in children. If DNA reservoirs contribute to the RNA pool recurrently, it appears relevant to consider both DNA and RNA sequences to avoid features of HIV-1 evolution. The potential bias introduced by the maternal sequences must be considered also. They were issued from cellular DNA since RNA sequences were not available for most mothers as a consequence of the low plasma viral load at delivery in response to antiretroviral prophylaxis during pregnancy [31]. However, the inferred TMRCA using RNA-derived sequence only were still consistent with the full analysis when considering the seven mother-infant pairs for which the number of sequences issued from plasma RNA was sufficient. Sampling times lead to a local rescaling of rates of substitutions through the local clock model and branching. Even if it constraints the relative nodes positions along the trees, the time estimate of the child's root provided confidence in the TMRCA inference and was confirmed by the congruence with the biomedical assays. Another limitation may be linked to the technical strategy used. Indeed, the sequences were not obtained using the single genome amplification technology that is considered as a gold standard since it avoid recombination events during the PCR from bulk DNA and it limits the non proportional representation of target sequences due to template resampling [60]–[62]. However, and as discussed above, the concordance between the molecular evolution approach and the reference biomedical diagnosis suggest that this limitation did not have a drastic effect.

## Conclusions

This study has benefited from the temporal anchoring of HIV-1 sequences to infer transmission timing and evolutionary rates in mother-infant pairs. Thanks to the validation based on the determination of the transmission period through the reference diagnostic procedure, our study offered a unique opportunity to validate the Bayesian coalescent framework on documented cases. It confirms that time series of viral sequences can provide powerful insights to draw inference about transmission timing. Such theoretical approaches could also provide insight into multiple infections processes (i.e. coinfection or superinfection) not readily detectable with the current diagnostic assays.

## Supporting Information

Table S1 **Bayes factor of the strict molecular clock (M1) and the uncorrelated lognormal clock (M0) models for each pair.** Bayesian Analysis were conducted under the best-fitting set of molecular, coalescent and demographic models as indicated by a Bayes Factor (*B_01_*)>20
(DOCX)

## References

[1] Prendergast A, Tudor-Williams G, Jeena P, Burchett S, Goulder P (2007) International perspectives, progress, and future challenges of paediatric HIV infection. Lancet 370: 68–80 10.1016/S0140-6736(07)61051-4 17617274

[2] Rouzioux C, Costagliola D, Burgard M, Blanche S, Mayaux MJ, et al (1995) Estimated timing of mother-to-child human immunodeficiency virus type 1 (HIV-1) transmission by use of a Markov model. The HIV Infection in Newborns French Collaborative Study Group. Am J Epidemiol 142: 1330–1337. 7503054 10.1093/oxfordjournals.aje.a117601

[3] Rambaut A, Posada D, Crandall KA, Holmes EC (2004) The causes and consequences of HIV evolution. Nat Rev Genet 5: 52–61 10.1038/nrg1246nrg1246pii 14708016

[4] Holmes EC, Nee S, Rambaut A, Garnett GP, Harvey PH (1995) Revealing the history of infectious disease epidemics through phylogenetic trees. Philos Trans R Soc Lond B Biol Sci 349: 33–40 10.1098/rstb.1995.0088 8748017

[5] Junqueira DM, de Medeiros RM, Matte MCC, Araújo LAL, Chies JAB, et al (2011) Reviewing the history of HIV-1: spread of subtype B in the Americas. PloS One 6: e27489 10.1371/journal.pone.0027489 22132104 PMC3223166

[6] Worobey M, Gemmel M, Teuwen DE, Haselkorn T, Kunstman K, et al (2008) Direct evidence of extensive diversity of HIV-1 in Kinshasa by 1960. Nature 455: 661–664 10.1038/nature07390 18833279 PMC3682493

[7] Edwards CTT, Holmes EC, Wilson DJ, Viscidi RP, Abrams EJ, et al (2006) Population genetic estimation of the loss of genetic diversity during horizontal transmission of HIV-1. BMC Evol Biol 6: 28 10.1186/1471-2148-6-28 16556318 PMC1444934

[8] Hué S, Pillay D, Clewley JP, Pybus OG (2005) Genetic analysis reveals the complex structure of HIV-1 transmission within defined risk groups. Proc Natl Acad Sci U S A 102: 4425–4429 10.1073/pnas.0407534102 15767575 PMC555492

[9] Lemey P, Rambaut A, Pybus OG (2006) HIV evolutionary dynamics within and among hosts. AIDS Rev 8: 125–140. 17078483

[10] Castro-Nallar E, Pérez-Losada M, Burton GF, Crandall KA (2012) The evolution of HIV: inferences using phylogenetics. Mol Phylogenet Evol 62: 777–792 10.1016/j.ympev.2011.11.019 22138161 PMC3258026

[11] Nickle DC, Jensen MA, Shriner D, Brodie SJ, Frenkel LM, et al (2003) Evolutionary Indicators of Human Immunodeficiency Virus Type 1 Reservoirs and Compartments. J Virol 77: 5540–5546 10.1128/JVI.77.9.5540-5546.2003 12692259 PMC153940

[12] Rachinger A, Stolte IG, van de Ven TD, Burger JA, Prins M, et al (2010) Absence of HIV-1 superinfection 1 year after infection between 1985 and 1997 coincides with a reduction in sexual risk behavior in the seroincident Amsterdam cohort of homosexual men. Clin Infect Dis Off Publ Infect Dis Soc Am 50: 1309–1315 10.1086/651687 20367230

[13] Shankarappa R, Margolick JB, Gange SJ, Rodrigo AG, Upchurch D, et al (1999) Consistent viral evolutionary changes associated with the progression of human immunodeficiency virus type 1 infection. J Virol 73: 10489–10502. 10559367 10.1128/jvi.73.12.10489-10502.1999 PMC113104

[14] Zhu T, Wang N, Carr A, Nam DS, Moor-Jankowski R, et al (1996) Genetic characterization of human immunodeficiency virus type 1 in blood and genital secretions: evidence for viral compartmentalization and selection during sexual transmission. J Virol 70: 3098–3107. 8627789 10.1128/jvi.70.5.3098-3107.1996 PMC190172

[15] Drummond AJ, Ho SYW, Phillips MJ, Rambaut A (2006) Relaxed phylogenetics and dating with confidence. PLoS Biol 4: e88 10.1371/journal.pbio.0040088 16683862 PMC1395354

[16] Lewis F, Hughes GJ, Rambaut A, Pozniak A, Leigh Brown AJ (2008) Episodic sexual transmission of HIV revealed by molecular phylodynamics. PLoS Med 5: e50 10.1371/journal.pmed.0050050 18351795 PMC2267814

[17] Bernard EJ, Azad Y, Vandamme AM, Weait M, Geretti AM (2007) HIV forensics: pitfalls and acceptable standards in the use of phylogenetic analysis as evidence in criminal investigations of HIV transmission. HIV Med 8: 382–387 10.1111/j.1468-1293.2007.00486.x 17661846

[18] Poon AFY, McGovern RA, Mo T, Knapp DJHF, Brenner B, et al (2011) Dates of HIV infection can be estimated for seroprevalent patients by coalescent analysis of serial next-generation sequencing data. AIDS Lond Engl 25: 2019–2026 10.1097/QAD.0b013e32834b643c 21832936

[19] English S, Katzourakis A, Bonsall D, Flanagan P, Duda A, et al (2011) Phylogenetic analysis consistent with a clinical history of sexual transmission of HIV-1 from a single donor reveals transmission of highly distinct variants. Retrovirology 8: 54 10.1186/1742-4690-8-54 21736738 PMC3161944

[20] Kaye M, Chibo D, Birch C (2009) Comparison of Bayesian and maximum-likelihood phylogenetic approaches in two legal cases involving accusations of transmission of HIV. AIDS Res Hum Retroviruses 25: 741–748 10.1089/aid.2008.0306 19619011

[21] Rachinger A, Groeneveld PHP, van Assen S, Lemey P, Schuitemaker H (2011) Time-measured phylogenies of gag, pol and env sequence data reveal the direction and time interval of HIV-1 transmission. AIDS Lond Engl 25: 1035–1039 10.1097/QAD.0b013e3283467020 21505318

[22] Brossard Y, Aubin JT, Mandelbrot L, Bignozzi C, Brand D, et al (1995) Frequency of early in utero HIV-1 infection: a blind DNA polymerase chain reaction study on 100 fetal thymuses. AIDS Lond Engl 9: 359–366. 7794540

[23] Bryson YJ, Luzuriaga K, Sullivan JL, Wara DW (1992) Proposed definitions for in utero versus intrapartum transmission of HIV-1. N Engl J Med 327: 1246–1247 10.1056/NEJM199210223271718 1406816

[24] Gilbert MTP, Rambaut A, Wlasiuk G, Spira TJ, Pitchenik AE, et al (2007) The emergence of HIV/AIDS in the Americas and beyond. Proc Natl Acad Sci U S A 104: 18566–18570 10.1073/pnas.0705329104 17978186 PMC2141817

[25] Hughes GJ, Fearnhill E, Dunn D, Lycett SJ, Rambaut A, et al (2009) Molecular phylodynamics of the heterosexual HIV epidemic in the United Kingdom. PLoS Pathog 5: e1000590 10.1371/journal.ppat.1000590 19779560 PMC2742734

[26] Pybus OG, Rambaut A, Harvey PH (2000) An integrated framework for the inference of viral population history from reconstructed genealogies. Genetics 155: 1429–1437. 10880500 10.1093/genetics/155.3.1429 PMC1461136

[27] Drummond A, Pybus OG, Rambaut A (2003) Inference of viral evolutionary rates from molecular sequences. Adv Parasitol 54: 331–358. 14711090 10.1016/s0065-308x(03)54008-8

[28] Hudson RR (1983) Properties of a neutral allele model with intragenic recombination. Theor Popul Biol 23: 183–201. 6612631 10.1016/0040-5809(83)90013-8

[29] Grenfell BT, Pybus OG, Gog JR, Wood JLN, Daly JM, et al (2004) Unifying the epidemiological and evolutionary dynamics of pathogens. Science 303: 327–332 10.1126/science.1090727 14726583

[30] Rosenberg NA, Nordborg M (2002) Genealogical trees, coalescent theory and the analysis of genetic polymorphisms. Nat Rev Genet 3: 380–390 10.1038/nrg795 11988763

[31] Lallemant M, Jourdain G, Le Coeur S, Kim S, Koetsawang S, et al (2000) A trial of shortened zidovudine regimens to prevent mother-to-child transmission of human immunodeficiency virus type 1. Perinatal HIV Prevention Trial (Thailand) Investigators. N Engl J Med 343: 982–991. 11018164 10.1056/NEJM200010053431401

[32] Samleerat T, Braibant M, Jourdain G, Moreau A, Ngo-Giang-Huong N, et al (2008) Characteristics of HIV type 1 (HIV-1) glycoprotein 120 env sequences in mother-infant pairs infected with HIV-1 subtype CRF01_AE. J Infect Dis 198: 868–876 10.1086/591251 18700833

[33] Katoh K, Asimenos G, Toh H (2009) Multiple alignment of DNA sequences with MAFFT. Methods Mol Biol Clifton NJ 537: 39–64 10.1007/978-1-59745-251-93 19378139

[34] Waterhouse AM, Procter JB, Martin DMA, Clamp M, Barton GJ (2009) Jalview Version 2–a multiple sequence alignment editor and analysis workbench. Bioinforma Oxf Engl 25: 1189–1191 10.1093/bioinformatics/btp033 PMC2672624 19151095

[35] Excoffier L, Smouse PE, Quattro JM (1992) Analysis of molecular variance inferred from metric distances among DNA haplotypes: application to human mitochondrial DNA restriction data. Genetics 131: 479–491. 1644282 10.1093/genetics/131.2.479 PMC1205020

[36] Excoffier L, Laval G, Schneider S (2005) Arlequin (version 3.0): an integrated software package for population genetics data analysis. Evol Bioinforma Online 1: 47–50. PMC2658868 19325852

[37] Guindon S, Gascuel O (2003) A simple, fast, and accurate algorithm to estimate large phylogenies by maximum likelihood. Syst Biol 52: 696–704. 14530136 10.1080/10635150390235520

[38] Delport W, Poon AFY, Frost SDW, Kosakovsky Pond SL (2010) Datamonkey 2010: a suite of phylogenetic analysis tools for evolutionary biology. Bioinforma Oxf Engl 26: 2455–2457 10.1093/bioinformatics/btq429 PMC2944195 20671151

[39] Pond SLK, Frost SDW, Muse SV (2005) HyPhy: hypothesis testing using phylogenies. Bioinforma Oxf Engl 21: 676–679 10.1093/bioinformatics/bti079 15509596

[40] Kosakovsky Pond SL, Posada D, Gravenor MB, Woelk CH, Frost SDW (2006) Automated phylogenetic detection of recombination using a genetic algorithm. Mol Biol Evol 23: 1891–1901 10.1093/molbev/msl051 16818476

[41] Kishino H, Hasegawa M (1989) Evaluation of the maximum likelihood estimate of the evolutionary tree topologies from DNA sequence data, and the branching order in hominoidea. J Mol Evol 29: 170–179. 2509717 10.1007/BF02100115

[42] Drummond AJ, Suchard MA, Xie D, Rambaut A (2012) Bayesian Phylogenetics with BEAUti and the BEAST 1.7. Mol Biol Evol 29: 1969–1973 10.1093/molbev/mss075 22367748 PMC3408070

[43] Heled J, Drummond AJ (2008) Bayesian inference of population size history from multiple loci. BMC Evol Biol 8: 289 10.1186/1471-2148-8-289 18947398 PMC2636790

[44] Kass RE, Raftery AE (1995) Bayes Factors. J Am Stat Assoc 90: 773–795 10.1080/01621459.1995.10476572

[45] Kosakovsky Pond SL, Posada D, Gravenor MB, Woelk CH, Frost SDW (2006) GARD: a genetic algorithm for recombination detection. Bioinforma Oxf Engl 22: 3096–3098 10.1093/bioinformatics/btl474 17110367

[46] Ahmad N, Baroudy BM, Baker RC, Chappey C (1995) Genetic analysis of human immunodeficiency virus type 1 envelope V3 region isolates from mothers and infants after perinatal transmission. J Virol 69: 1001–1012. 7815476 10.1128/jvi.69.2.1001-1012.1995 PMC188669

[47] Briant L, Wade CM, Puel J, Brown AJ, Guyader M (1995) Analysis of envelope sequence variants suggests multiple mechanisms of mother-to-child transmission of human immunodeficiency virus type 1. J Virol 69: 3778–3788. 7745725 10.1128/jvi.69.6.3778-3788.1995 PMC189095

[48] Dickover RE, Garratty EM, Plaeger S, Bryson YJ (2001) Perinatal transmission of major, minor, and multiple maternal human immunodeficiency virus type 1 variants in utero and intrapartum. J Virol 75: 2194–2203 10.1128/JVI.75.5.2194-2203.2001 11160723 PMC114803

[49] Kishko M, Somasundaran M, Brewster F, Sullivan JL, Clapham PR, et al (2011) Genotypic and functional properties of early infant HIV-1 envelopes. Retrovirology 8: 67 10.1186/1742-4690-8-67 21843318 PMC3189118

[50] Kwiek JJ, Russell ES, Dang KK, Burch CL, Mwapasa V, et al (2008) The molecular epidemiology of HIV-1 envelope diversity during HIV-1 subtype C vertical transmission in Malawian mother-infant pairs. AIDS Lond Engl 22: 863–871 10.1097/QAD.0b013e3282f51ea0 PMC2760232 18427205

[51] Pasquier C, Cayrou C, Blancher A, Tourne-Petheil C, Berrebi A, et al (1998) Molecular evidence for mother-to-child transmission of multiple variants by analysis of RNA and DNA sequences of human immunodeficiency virus type 1. J Virol 72: 8493–8501. 9765386 10.1128/jvi.72.11.8493-8501.1998 PMC110258

[52] Renjifo B, Chung M, Gilbert P, Mwakagile D, Msamanga G, et al (2003) In-utero transmission of quasispecies among human immunodeficiency virus type 1 genotypes. Virology 307: 278–282. 12667797 10.1016/s0042-6822(02)00066-1

[53] Russell ES, Kwiek JJ, Keys J, Barton K, Mwapasa V, et al (2011) The genetic bottleneck in vertical transmission of subtype C HIV-1 is not driven by selection of especially neutralization-resistant virus from the maternal viral population. J Virol 85: 8253–8262 10.1128/JVI.00197-11 21593171 PMC3147968

[54] Scarlatti G, Leitner T, Halapi E, Wahlberg J, Jansson M, et al (1993) Analysis of the HIV-1 envelope V3-loop sequences from ten mother-child pairs. Ann N Y Acad Sci 693: 277–280. 8267278 10.1111/j.1749-6632.1993.tb26282.x

[55] Wolinsky SM, Wike CM, Korber BT, Hutto C, Parks WP, et al (1992) Selective transmission of human immunodeficiency virus type-1 variants from mothers to infants. Science 255: 1134–1137. 1546316 10.1126/science.1546316

[56] Zhang H, Tully DC, Hoffmann FG, He J, Kankasa C, et al (2010) Restricted genetic diversity of HIV-1 subtype C envelope glycoprotein from perinatally infected Zambian infants. PloS One 5: e9294 10.1371/journal.pone.0009294 20174636 PMC2823783

[57] Lee HY, Giorgi EE, Keele BF, Gaschen B, Athreya GS, et al (2009) Modeling sequence evolution in acute HIV-1 infection. J Theor Biol 261: 341–360 10.1016/j.jtbi.2009.07.038 19660475 PMC2760689

[58] Shen L, Siliciano RF (2008) Viral reservoirs, residual viremia, and the potential of highly active antiretroviral therapy to eradicate HIV infection. J Allergy Clin Immunol 122: 22–28 10.1016/j.jaci.2008.05.033 18602567 PMC6812482

[59] Zhang J, Nielsen R, Yang Z (2005) Evaluation of an improved branch-site likelihood method for detecting positive selection at the molecular level. Mol Biol Evol 22: 2472–2479 10.1093/molbev/msi237 16107592

[60] Lahr DJG, Katz LA (2009) Reducing the impact of PCR-mediated recombination in molecular evolution and environmental studies using a new-generation high-fidelity DNA polymerase. BioTechniques 47: 857–866 10.2144/000113219 19852769

[61] Eckert KA, Kunkel TA (1991) DNA polymerase fidelity and the polymerase chain reaction. PCR Methods Appl 1: 17–24. 1842916 10.1101/gr.1.1.17

[62] Horton RM (1995) PCR-mediated recombination and mutagenesis. SOEing together tailor-made genes. Mol Biotechnol 3: 93–99 10.1007/BF02789105 7620981

