# Supplementary material for: Estimating the Timing of Mother-to-Child Transmission of the Human Immunodeficiency Virus Type 1 Using a Viral Molecular Evolution Model
Source: PLoS One. 2014 Apr 9;9(4):e90421. doi: 10.1371/journal.pone.0090421 (PMC3981669; doi:10.1371/journal.pone.0090421)
Supplement: Table S1 — Bayes factor of the strict molecular clock (M1) and the uncorrelated lognormal clock (M0) models for each pair. Bayesian Analysis were conducted under the best-fitting set of molecular, coalescent and demographic models as indicated by a Bayes Factor (B01)>20 (DOCX) [file pone.0090421.s001.docx]

**Table S1 - Bayes factor of the strict molecular clock (M1) and the uncorrelated lognormal clock (M0) models for each pair.**

| **Pairs** | **ln f(X/*M_1_*)** | **ln f(X/*M_0_*)** | **2ln*B_01_*** | **Evidence against *M_1_*** | **Selected model** |
| --- | --- | --- | --- | --- | --- |
| **0779** | -4825,20 (+/- 0,23) | -4786,18 (+/- 0,28) | 78,04 | Very strong | *M_0_* |
| **0858** | -4318,20 (+/- 0,23) | -4279,84 (+/- 0,30) | 76,91 | Very strong | *M_0_* |
| **0939** | -4234,24 (+/- 0,19) | -4207,77 (+/- 0,23) | 52,94 | Very strong | *M_0_* |
| **1005** | -8131,29 (+/- 0,28) | -8005,66 (+/- 0,35) | 251,26 | Very strong | *M_0_* |
| **1021** | -4385,93 (+/- 0,20) | -4329,35 (+/- 0,19) | 113,16 | Very strong | *M_0_* |
| **1110** | -3653,01 (+/- 0,13) | -3627,16 (+/- 0,16) | 51,70 | Very strong | *M_0_* |
| **1224** | -4043,53 (+/- 0,21) | -4039,92 (+/- 0,23) | 7,22 | Strong | *M_0_* |
| **1333** | -4691,34 (+/- 0,19) | -4642,20 (+/- 0,20) | 98,27 | Very strong | *M_0_* |
| **1391** | -4956,62 (+/- 0,23) | -4918,73 (+/- 0,23) | 75,77 | Very strong | *M_0_* |

M_1_ model: strict molecular clock; M_0_ model: uncorrelated lognormal (relaxed) clock; ln: logarithm; B_01_: Bayes Factor.

Note: The *Bayes factor* (*B_01_*) is the ratio of the likelihoods of the two models, f(X/*M_0_*)/f(X/*M_1_*). The critical values for Bayes factors comparisons come from the Kass & Raftery [44].
